# Seroprevalence of TTIs and its association with ABO and Rh blood groups among blood donors

**DOI:** 10.6026/973206300211912

**Published:** 2025-07-31

**Authors:** Jyoti Kala Bharati, Nouratan Singh, Shweta Chaudhary, Aaditya Shivhare, Arvind Kumar Singh, Yatendra Mohan

**Affiliations:** 1 Department of Transfusion Medicine, Uttar Pradesh University of Medical Sciences, Saifai, Etawah, Uttar Pradesh-206130, India

**Keywords:** Chemiluminescent immunoassays (CLIA), Nucleic Acid Testing (NAT), Transfusion-Transmitted Infections (TTI), ABO/Rh blood groups, seroprevalence, blood donors, chi-square test

## Abstract

Transfusion-transmitted infections (TTIs) such as HIV, HBV, HCV, syphilis, and malaria pose significant risks to blood safety. Therefore, it is of interest to assess the seroprevalence of TTIs and their association with ABO/Rh blood groups among 30,335 blood donors. Data from 30,335 donations, with 1,843 reactive units, were analyzed demographically. Overall TTI seroprevalence was 6.08%. O Negative showed the highest reactive rate (9.41%, OR=1.62, 95% CI: 1.14-2.30), followed by B Positive (7.06%, OR=1.26, 95% CI: 1.14-1.39). AB Positive had the lowest rate (4.28%, OR=0.66, 95% CI: 0.56-0.78). A significant association was confirmed (χ^2^=53.9, df=7, p<0.0001). O Negative and B Positive blood groups are strongly associated with higher TTI seroprevalence, while AB Positive shows lower risk. Thus, targeted screening for high-risk groups could enhance blood safety.

## Background:

Blood transfusion is a critical intervention but carries risks of transfusion-transmitted infections (TTIs), including HIV, hepatitis B virus (HBV), hepatitis C virus (HCV), syphilis, and malaria [1]. HBV, HCV, and HIV are particularly severe, often causing chronic, life-altering infections [2]. Despite stringent donor screening, TTI prevalence varies by donor demographics and regional disease patterns [3]. Recent studies suggest ABO and Rh blood groups may modulate susceptibility to infectious diseases, potentially affecting TTI seroprevalence in blood donors [4, 5]. For instance, certain blood groups have been linked to differential risks for viral hepatitis and parasitic infections [6]. The World Health Organization (WHO) recommends mandatory screening of blood donors for these infections to reduce the risk of transmission [7]. While developed countries have successfully implemented advanced screening techniques, resource constraints in developing countries result in a higher incidence of TTIs during blood transfusions [8]. The ABO and Rh blood group systems are not only essential for safe blood transfusions but also have potential links to susceptibility to various infections, including TTIs [9]. The ABO blood group system, discovered by Karl Landsteiner in 1901, classifies individuals into four groups: A, B, AB, and O, based on antigens on the red blood cells [10]. The Rh system further classifies individuals as Rh-positive or Rh-negative [11]. Recent studies suggest that ABO and Rh blood groups may influence TTI susceptibility, but findings are inconsistent, especially in regions like Uttar Pradesh, where data remain limited [12]. Therefore, it is of interest to report the seroprevalence of transfusion-transmitted infections (TTIs) among blood donors in the southwestern portion of Uttar Pradesh and to investigate the potential association between ABO and Rh blood groups and TTI prevalence.

## Materials and Methods:

## Study design and period:

Retrospective study, January 2022-December 2024. Blood Centre, Department of Transfusion Medicine, Uttar Pradesh University of Medical Sciences, Saifai, Etawah, U.P., India.

## Study population and eligibility criteria:

## Eligible blood donors per National Blood Bank criteria:

Age: 18-65 years,

Weight: >45 kg,

Hemoglobin: >12.5 g/dL, Passed donor screening (questionnaire, physical exam).

Data collected: name, age, sex, blood group, address, donation type, frequency, TTI results.

## Laboratory tests:

## Blood grouping:

Micro-agglutination and gel matrix methods for ABO/Rh typing, confirmed by forward/reverse grouping [13].

## TTI screening:

Chemiluminescent immunoassays (CLIA) and NAT for HBV (HBsAg), HCV (anti-HCV), HIV (HIV-1/2 Ag/Ab), Syphilis (Treponema pallidum) using Abbott Architect 1000i SR. Malaria tested via rapid cards.

## NAT testing:

Confirmed HBV, HCV, HIV [14].

## Data analysis:

Calculate TTI and blood group prevalence. Chi-square tests and logistic regression assessed ABO/Rh-TTI associations (p < 0.05) [15].

## Results:

The analysis of 30,335 blood donations reveals critical insights into donation distributions, transfusion-transmitted infection (TTI) reactivity rates, and their implications for blood safety. B Positive (28.77%), O Positive (28.15%), and A Positive (25.54%) dominate, contributing ~82% of donations, reflecting their prevalence in the studied population, while rare blood groups like AB Negative (0.41%), O Negative (1.30%), and A Negative (1.24%) have minimal shares, consistent with their rarity. Analysis of 30,335 blood donors reveals 95.27% male (28,901) and 4.73% female (1,434) participation, indicating gender disparity etc. shown in (Table 1 - see PDF). First-time donors comprise 67% (20,315), repeat donors 33% (10,020). Voluntary donations dominate at 88.84% (26,950), with replacement at 11.16% (3,385) shown in (Figure 1 - see PDF). Age distribution shows 48.9% (14,835) aged 18-30, 26.5% (8,045) aged 31-40, 14.6% (4,425) aged 41-50, and 10% (3,030) of aged group 51-60, shown in (Figure 2 - see PDF). Health and medical workers lead occupations at 37.7% (11,450), followed by private job/business (29.8%, 9,030), students (16.6%, 5,035), and farmers (15.9%, 4,820) shown in (Figure 3 - see PDF). Reactivity affects 6.08% (1,843) of donors, with males at 97.45% (1,796) and females 2.55% (47) of reactive cases. CLIA detects 1,760 cases, NAT 83. Male reactivity is 5.92%, female 0.15% shown in (Table 1 - see PDF). Low female participation suggests barriers; high voluntary donations reflect community support; first-time donor prevalence requires retention focus. The analysis of 30,335 blood donations screened for transfusion-transmitted infections (TTIs), 1,843 were reactive, yielding an overall seroprevalence of 6.08% (1,843/30,335). The distribution of donations and reactive units across ABO & Rh blood groups is presented in (Table 2 - see PDF and Figure 4 - see PDF). Comparison of donation and reactive proportions, the overall TTI reactivity rate is 6.08% (1,843 reactive units), with O Negative 9.41% (37/393), AB Negative 7.20% (9/125), B Positive 7.06% (616/8729), and B Negative 7.00% (38/543) exceeding the average, indicating higher TTI prevalence, whereas AB Positive 4.28% (166/3879) and A Positive 5.19% (402/7749) fall below, suggesting lower risk shown in (Figure 5 - see PDF). The remaining were indicated average TTI prevalence in O positive 6.48% (553/8540) and A Negative 5.84% (22/377) shown in (Table 2 - see PDF) shown in (Figure 6 - see PDF). Notably, rare blood groups like O Negative and AB Negative show higher reactive proportions despite smaller donation shares, while common groups like A Positive and AB Positive exhibit lower reactivity. The Difference (%) metric highlights disparities, O Negative (+8.11%) and AB Negative (+6.79%) contributes disproportionately to reactive units, with O Negative's universal donor status raising concerns for blood safety, and B Negative (+5.21%) and A Negative (+4.60%) also show elevated reactivity. Conversely, B Positive (-21.71%), O Positive (-21.68%), A Positive (-20.35%), and AB Positive (-8.50%) have lower reactive proportions, with AB Positive's low reactivity suggesting a protective effect shown in (Table 3 - see PDF). Biologically, variations in reactivity may stem from genetic differences in immune response or antigen expression, such as O Negative's lack of A, B, and Rh antigens potentially increasing susceptibility to infections like hepatitis or HIV, while AB Positive's unique antigen profile may reduce TTI risk. A chi-square test (statistic = 54.91, df = 7, p < 0.0001) confirms a significant association between ABO/Rh blood groups and TTI reactivity. Major contributors include AB Positive (20.66, lower reactivity: 166 vs. 235.87 expected), B Positive (13.70, higher reactivity: 616 vs. 530.77 expected), A Positive (10.11, lower reactivity: 402 vs. 471.02 expected), and O Negative (7.19, higher reactivity: 37 vs. 23.89 expected). Smaller contributions from A Negative (0.04) and AB Negative (0.26) indicate reactivity near expected values shown in (Table 4 - see PDF). Odds ratios (OR) further clarify risk. O Negative (OR = 1.90, 95% CI: 1.33-2.71) has nearly twice the TTI reactivity odds compared to A Positive. AB Negative (OR = 1.42, 95% CI: 0.71-2.84) shows elevated but non-significant odds due to small sample size. B Positive (OR = 1.39, 95% CI: 1.21-1.59) and O Positive (OR = 1.26, 95% CI: 1.10-1.45) indicate significant increased risk, while B Negative (OR = 1.38, 95% CI: 0.97-1.95) and A Negative (OR = 1.13, 95% CI: 0.73-1.76) are non-significant. AB Positive (OR = 0.82, 95% CI: 0.68-0.98) suggests a protective effect. These findings highlight O Negative and B Positive as high-risk groups and AB Positive as lower-risk, aligning with chi-square results shown in (Table 5 - see PDF).

## Discussion:

Analysis of 30,335 blood donors reveals 95.27% male (28,901) and 4.73% female (1,434) participation, indicating gender disparity [17]. First-time donors comprise 67% (20,315), while repeat donors account for 33% (10,020) [18]. Voluntary donations dominate at 88.84% (26,950), with replacement donations at 11.16% (3,385) [19]. Age distribution shows 48.9% (14,835) aged 18-30, 26.5% (8,045) aged 31-40, 14.6% (4,425) aged 41-50, and 10% (3,030) aged 51-60 [20]. Health and medical workers lead occupations at 37.7% (11,450), followed by private job/business (29.8%, 9,030), students (16.6%, 5,035), and farmers (15.9%, 4,820) [21]. Reactivity affects 6.07% (1,843) of donors, with males at 97.45% (1,796) and females 2.55% (47) of reactive cases [22]. Chemiluminescence Immunoassay (CLIA) detects 1,760 cases, while Nucleic Acid Testing (NAT) identifies 83 [23]. Male reactivity is 5.92%, and female reactivity is 0.15% [24]. Low female participation suggests barriers; high voluntary donations reflect community support; and first-time donor prevalence indicates a need for retention focus [25]. The overall TTI reactivity rate is 6.08% (1,843 reactive units), with O Negative 9.41% (37/393), AB Negative 7.20% (9/125), B Positive 7.06% (616/8729), and B Negative 7.00% (38/543) exceeding the average, indicating higher TTI prevalence, whereas AB Positive 4.28% (166/3879) and A Positive 5.19% (402/7749) fall below, suggesting lower risk [26]. The rare blood groups like O Negative and AB Negative show higher reactive proportions despite smaller donation shares, while common groups like A Positive and AB Positive exhibit lower reactivity [27]. The difference (%) metric reveals significant disparities in blood reactivity: O Negative (+8.11%) and AB Negative (+6.79%) exhibit the highest rates, with O Negative's universal donor status posing potential blood safety risks. B Negative (+5.21%) and A Negative (+4.60%) also demonstrate notable reactivity [28]. Conversely, B Positive (-21.71%), O Positive (-21.68%), A Positive (-20.35%), and AB Positive (-8.50%) have lower reactive proportions, with AB Positive's low reactivity suggesting a protective effect [11]. Biologically, variations in reactivity may stem from genetic differences in immune response or antigen expression, such as O Negative's lack of A, B, and Rh antigens potentially increasing susceptibility to infections like hepatitis or HIV, while AB Positive's unique antigen profile may reduce TTI risk [5, 29]. Blood group prevalence differs across ethnicities and regions, possibly linked to TTI risk factors and behavioral variations in donation frequency or exposure, warranting further investigation [20, 21, 30].

## Conclusion:

O Negative and B Positive blood groups are strongly associated with higher TTI seroprevalence, with reactive rates of 9.41% (OR=1.90) and 7.06% (OR=1.39), respectively. We found prevalence of TTIs and association with blood group varies by demographically. The disproportionate reactivity of rare blood groups may limit their availability, necessitating strategic stockpiling, while donor education and regional health policies tailored to TTI prevalence by blood group can enhance safety. Hence, it is recommended to (1) Implement enhanced screening protocols for O Negative and B Positive donors; (2) Conduct studies with infection-specific data in larger samples for rare blood groups.
